# Necessity for retrospective evaluation of past-positive chemicals in in vitro chromosomal aberration tests using recommended cytotoxicity indices

**DOI:** 10.1186/s41021-017-0091-y

**Published:** 2018-01-10

**Authors:** Hiroshi Honda, Yurika Fujita, Toshio Kasamatsu, Anne Fuchs, Rolf Fautz, Osamu Morita

**Affiliations:** 1 R&D Safety Science Research, Kao Corporation, 2606 Akabane, Ichikai–Machi, Haga–Gun, Tochigi, 321–3497 Japan; 2 Kao Europe Research Laboratories, Kao Germany GmbH, Darmstadt, 64297 Germany

**Keywords:** Chromosomal aberration, Retrospective evaluation, Cytotoxicity index, In vitro genotoxicity test

## Abstract

We have demonstrated that retrospective evaluation of existing data of in vitro chromosomal aberration test using the new cytotoxicity indices RICC (relative increase in cell count) or RPD (relative population doubling) reduces the false-positive rate. We have constructed an algorithm to predict the likelihood that past-positive results would differ when retested accordingly. Here, we emphasize the importance of reviewing existing in vitro chromosomal aberration test results. The present Letter not only supports the rediscovery of potentially useful chemicals excluded from further development as a result of misclassification due to in vitro false-positive results, but also contributes to the development of a precise Quantitative Structure-Activity Relationship (QSAR) model by providing an appropriate training data-set. Furthermore, re-evaluation is expected to provide novel insights into underlying mechanisms and/or key structures involved in the development of chromosomal aberrations.

This letter is associated with the presentation entitled “Environmental mutagenesis and genomics research driven by big data and algorithms” at the JEMS Symposium on 10th June, 2017 [1]. In this letter, we would like to encourage researchers and/or institutions that have databases of in vitro chromosomal aberration testing data to review existing test results. Retrospective evaluation by replacing the original cytotoxicity index, relative cell counts (RCC), with the current standard indices, Relative Population Doubling (RPD) or Relative Increase in Cell Counts (RICC) is feasible using a mathematical method.

In vitro mammalian cell genotoxicity testing has been widely used for sensitive prediction of genotoxicity [2]. However, the application of this test remains controversial owing to the high rate of false-positive results generated under in vitro conditions [3]. The endpoints of chromosomal aberrations, which are linked to somatic mutations, have recently attracted much attention [4, 5], and are used in the in vivo micronucleus test that has not been criticized in regulatory sciences [6]. Moreover, in vitro genotoxicity tests possess the advantages of enabling the detection of non-DNA-binding genotoxic substances that act on chromosomes without the necessity for experimental animals [7]. Thus, in vitro mammalian genotoxicity testing may still be useful for evaluation of genotoxicity if the incidence of false positives may be reduced to within an acceptable range.

It is thought that severe cytotoxicity contributes to false positives in in vitro genotoxicity tests [8]. Moreover, non-physiological conditions that strongly inhibit cell division lead to irrelevant genotoxicity that resulted in false-positives [9]. Therefore, OECD test guidelines recommend the use of cytotoxicity indices such as RPD or RICC that take cell cycles into consideration, instead of RCC [10, 11]. The adoption of these new indices, as recommended by the OECD test guidelines, is expected to reduce the number of false positives generated by in vitro genotoxicity tests. Furthermore, some substances that have previously been falsely determined to be genotoxic may also be reclassified.

Accordingly, we attempted to replace past cytotoxicity indices with the current indices and constructed an algorithm to predict the likelihood that the test results would change when the new indices were adopted [12]. We retrospectively applied this algorithm to evaluate 129 substances in the Japanese database [13]. Of the 39 past-positive substances suggesting false-positive results, at least 11 showed negative results when re-analyzed [14]. In our review, retrospective evaluation had less impact on increase of false negatives. We confirmed it through review of the Ames test and in vivo micronucleus test results on the chemicals which were newly predicted as negatives. Review of other genotoxicity test results or in silico evaluation would be important for validations of false positives or false negatives identified. Thus, we conclude that our method should be useful for the effective re-classification of substances originally identified as positive in genotoxicity assays that are more likely to be negative.

There are two main reasons underlying the necessity for reviewing in vitro chromosomal aberration test results: First, such a review will contribute towards the rediscovery of useful chemical substances, particularly in the cosmetics industry where the EU prohibits animal experiments. They are not allowed conducting in vivo micronucleus testing to follow-up in vitro positive results. Our approach should enable the assessment of whether the initial test results are valid without animal testing. When you would like to know whether a substance with past positive results in in vitro tests and negative results in Ames tests may be redeveloped, we recommend replacing the original cytotoxicity indices with the current standards. This step should allow assessment of the possibility that the substance of interest will produce negative results in retests. We anticipate that the present method will enable the identification of substances with a high likelihood of negative results when clear toxic effects are recognized. We expect that, in some cases, this will hold true even without retesting in the context of regulatory acceptance. This approach will allow us to reassess compounds for which development has been halted because of difficulties in interpretation of genotoxicity results; we anticipate that the application of the present method should enable some of these compounds to re-enter development.

Second, a review of in vitro genotoxicity test results should contribute to the development of a high-precision Quantitative Structure-Activity Relationship (QSAR) model. Existing QSAR tools have been constructed based on databases that include past false-positive results. Therefore, there is an issue regarding the prediction of positive results, which should have been detected. We have previously reported the construction of a highly precise QSAR model by reviewing the results of training data [15]. It is hoped that all positive data contributing to the construction of QSAR models may be reevaluated using cytotoxicity indices. However, since the raw data are not publicly available in some large-scale chromosomal aberration test databases, such as NTP [16] and Snyder et al. (2004) [17], RCC values cannot be utilized. Therefore, the discovery and maintenance of the RCC values of these databases play an important role in the construction of a high-precision QSAR model. We hope this Letter will prompt global retrospective evaluations following the review of cytotoxicity indices of chromosomal aberration test results.

The present transformation theory of cytotoxicity indices should be useful for the refinement of genotoxicity evaluations that utilize readily available chromosomal aberration test results. We wish to emphasize that the true value of our technology does not lie in its ability to review judgments through manipulation of numerical values, but in its utility as a tool in genotoxicity evaluation to investigate the role of cell cycle inhibition and identify important chemical structures in chromosomal aberrations.

## Abbreviations

EU: European Union
JEMS: Japanese Environmental Mutagen Society
OECD: The Organisation for Economic Co-operation and Development
QSAR: Quantitative Structure-Activity Relationship
RCC: Relative cell count
RICC: Relative Increase in Cell Count
RPD: Relative Population Doubling
